# Optogenetic‐mediated cardiovascular differentiation and patterning of human pluripotent stem cells

**DOI:** 10.1002/ggn2.202100011

**Published:** 2021-09-10

**Authors:** Peter B. Hellwarth, Yun Chang, Arundhati Das, Po‐Yu Liang, Xiaojun Lian, Nicole A. Repina, Xiaoping Bao

**Affiliations:** ^1^ Davidson School of Chemical Engineering, Purdue University Center for Cancer Research Purdue University West Lafayette Indiana USA; ^2^ Department of Biomedical Engineering, Huck Institutes of the Life Sciences, Department of Biology Pennsylvania State University University Park Pennsylvania USA; ^3^ Friedrich Miescher Institute for Biomedical Research (FMI) Basel Switzerland

**Keywords:** cardiac differentiation and patterning, genome editing, human pluripotent stem cells, optogenetics, Wnt signaling

## Abstract

Precise spatial and temporal regulation of dynamic morphogen signals during human development governs the processes of cell proliferation, migration, and differentiation to form organized tissues and organs. Tissue patterns spontaneously emerge in various human pluripotent stem cell (hPSC) models. However, the lack of molecular methods for precise control over signal dynamics limits the reproducible production of tissue patterns and a mechanistic understanding of self‐organization. We recently implemented an optogenetic‐based OptoWnt platform for light‐controllable regulation of Wnt/β‐catenin signaling in hPSCs for *in vitro* studies. Using engineered illumination devices to generate light patterns and thus precise spatiotemporal control over Wnt activation, here we triggered spatially organized transcriptional changes and mesoderm differentiation of hPSCs. In this way, the OptoWnt system enabled robust endothelial cell differentiation and cardiac tissue patterning *in vitro*. Our results demonstrate that spatiotemporal regulation of signaling pathways via synthetic OptoWnt enables instructive stem cell fate engineering and tissue patterning.

## Graphical abstract

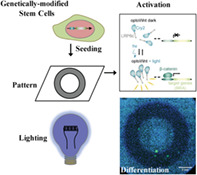

## 1 INTRODUCTION

Human pluripotent stem cells (hPSCs) possess great potential in developmental biology studies, regenerative medicine, disease modeling, and drug screening. For example, functional cardiomyocytes^1^, ^2^ (CMs) and cardiac organoids^3^, ^4^ have been recently produced from hPSCs via temporal regulation of Wnt signaling for the elucidation of human heart development. However, a broader application of hPSCs is currently limited by inability to create more physiologically relevant models that recapitulate the cell diversity and spatial organization of tissues. Precise spatial and temporal control of developmental pathways within an initially homogenous cell population would direct differentiation to generate organized patterns of heterogeneous cell populations and better recapitulate *in vivo* tissue structures. These heterogeneous cultures would permit cross cell‐type interactions that do not occur in mono‐cultures, allowing for synergistic maturation in cell phenotypes and more translatable models.^5^ For instance, micropatterning technology has been developed to construct spatially‐patterned cardiac organoids for developmental biology study and toxicity testing.^4^, ^6^

The light‐responsive optogenetic system OptoWnt^7^, ^8^ attains the desired precise spatial and temporal control representative of an ideal synthetic signaling construct. OptoWnt is composed of the photolyase homology domain of the *Arabidopsis thaliana* blue‐light photoreceptor cryptochrome 2 (Cry2) conjugated to the cytoplasmic fragment of the Wnt co‐receptor LRP6 (LRP6c).^9^ The complexity of differentiation obtainable using current differentiation protocols based on diffusible Wnt agonists is limited to temporal control of the soluble morphogen. By using a specialized LED device for light illumination at variable amplitudes (LAVA) and photomasks, our synthetic OptoWnt system can provide cell cultures with spatiotemporal control of Wnt signaling that was previously unachievable, enabling us to direct hPSC differentiation into complex heterogeneous cultures that recapitulate human early embryonic development.^8^

To demonstrate the application of this optogenetic system in stem cell fate engineering and tissue patterning, we first knocked the Cry2‐LRP6c and P2A‐mCherry conjugated OptoWnt construct into the *AAVS1* safe harbor locus via CRISPR/Cas9‐mediated homologous recombination. The resulting genetically modified OptoWnt hPSCs could activate canonical Wnt signaling in response to blue light illumination without any external morphogen treatment. Our previous work has demonstrated the important roles of Wnt signaling during cardiovascular differentiation,^2^, ^10^ and we show here that light‐induced Wnt activation could also differentiate hPSCs into endothelial progenitor cells (EPCs), epicardial cells, and CMs, with efficiencies comparable to classical morphogen‐induced differentiation approaches. For the first time, we precisely achieved spatially‐patterned mesoderm and cardiac tissues in monolayer cell cultures via light regulation, making OptoWnt a suitable tool for the precise engineering of tissue morphogenesis and organ development from hPSCs.

## 2 RESULTS

### 2.1 Design of a genetically engineered hPSC line for optogenetic Wnt activation

**FIGURE 1:**
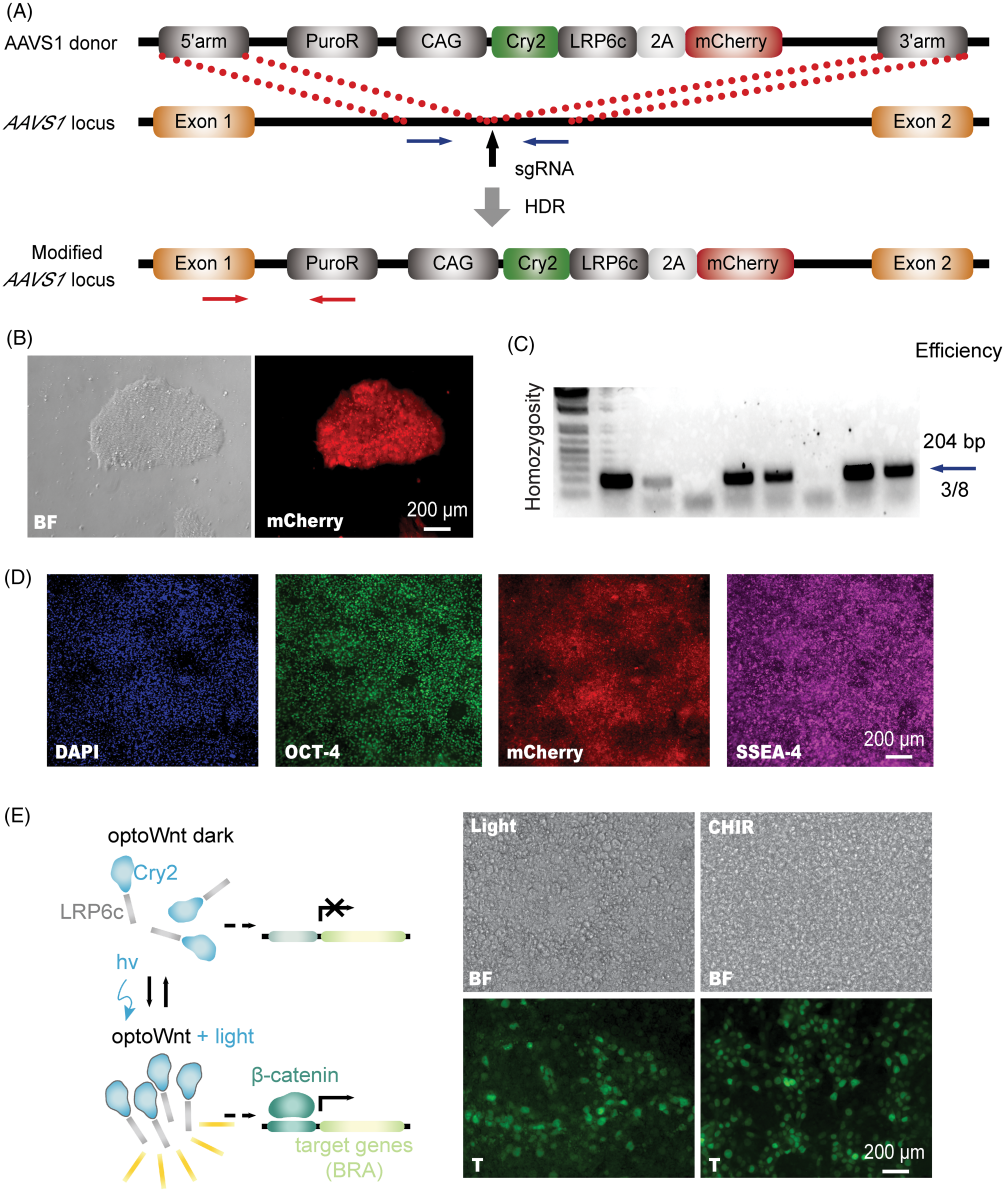
Construction of a genetically engineered human pluripotent stem cell (hPSC) line to activate Wnt signaling via optogenetics. (A) OptoWnt knock‐in schematic using CRISPR‐mediated homology directed repair at *AAVS1* safe harbor locus. (B) Brightfield (BF) and mCherry images of a successful OptoWnt knock‐in to generate a hPSC clonal line. (C) Homozygosity assay determining which clones have homozygous transgene insertion into both *AAVS1* alleles. Clones without ~200 bp PCR products were homozygous (blue arrow). (D) Representative images of immunostaining for OCT‐4, SSEA‐4 and mCherry in a homozygous OptoWnt clone. (E) Schematic of light‐induced Wnt activation and representative images of brightfield (BF) and T immunostaining in OptoWnt hPSCs after 48 hours illumination (Light) or 6 μM CHIR99021 (CHIR) treatment. Scale bars, 200 μm

The canonical Wnt signaling pathway is initiated when Wnt ligands bind to the transmembrane receptor Frizzled, leading to the clustering of LRP6, a Wnt co‐receptor.^11^ The formation of clusters triggers the phosphorylation of LRP6 and induces the downstream signaling cascade to stabilize β‐catenin, which is translocated to the nucleus and activates Wnt target genes, including mesoderm marker brachyury (or T).^12^ A synthetic optogenetic system, where the Cry2 photoreceptor was conjugated to the LRP6 (LRP6c) cytoplasmic domain (Figure 1A), was previously developed by Bugai *et al* ^9^ to allow for light‐inducible Wnt signaling.

To achieve stable expression of OptoWnt in hPSCs for light‐induced activation of Wnt, we firstly knocked the Cry2‐LRP6c‐mCherry transgene into the endogenous *AAVS1* safe harbor locus^13^ (Figure 1A) via CRISPR/Cas9‐mediated homologous recombination. The employment of P2A‐linked mCherry (2A‐mCherry) enabled the rapid identification and isolation of successfully targeted clones (Figure 1B), which were subjected for further homozygosity analysis (Figure 1C). The resulting OptoWnt hPSCs retained a pluripotent phenotype with expression of OCT4 and SSEA4 (Figure 1D). Since Wnt activation induces hPSC differentiation into brachyury^+^ (or T^+^) mesoderm,^14^, ^15^ we measured the percentage of T^+^ cells post‐illumination to optimize the blue light intensity using a LAVA illumination device^8^ (Figure S1A,B). After 48 hours of light illumination, the percentages of T^+^ cells were proportional to blue light intensity and approached saturation above 1 µW/mm^2^, yielding ~90% T^+^ cells and comparable to the positive control using a Wnt agonist CHIR99021 (CHIR, 6 μM) (Figure 1E, Figure S2). Our results demonstrated the feasibility of using light illumination to manipulate Wnt signaling in hPSCs for cell fate engineering and patterning.

### 2.2 Light‐induced EPC differentiation of hPSCs

**FIGURE 2:**
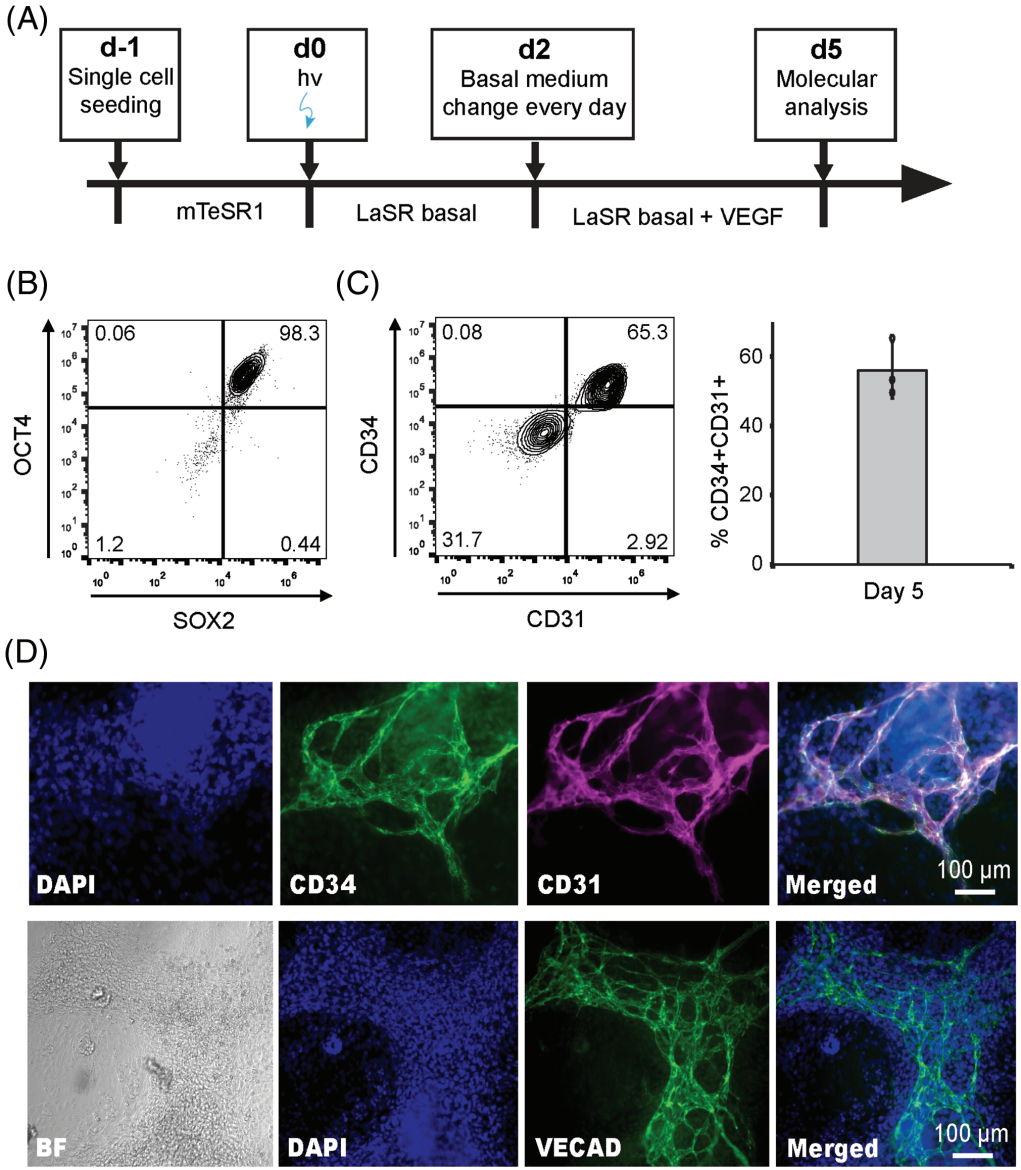
Optogenetic directed differentiation of human pluripotent stem cells (hPSCs) to endothelial progenitor cells (EPCs). (A) Protocol for differentiation of hPSCs to EPCs via OptoWnt light activation. (B,C) Representative flow cytometry analysis for expression of pluripotency markers OCT4 and SOX2 at day −2, and (C) EPC markers CD34 and CD31 at day 5 of the differentiation protocol. Quantification of CD34^+^CD31^+^ cells at day 5 is also shown. Data are represented as mean ± SD of three independent replicates. (D) Representative images of immunostaining for CD31, CD34, and VECAD in day 5 cultures. Scale bars, 100 μm

We further differentiated hPSCs into EPCs by replacing our previous CHIR‐based protocol^16^ with the light‐activated OptoWnt system (Figure 2A), which allows for spatial and temporal control over differentiation. Before light illumination, OptoWnt cells retained pluripotency as indicated by flow cytometry analysis of 98.1% OCT4^+^SOX2^+^ cells in hPSC culture (Figure 2B). After 5 days of continuous OptoWnt stimulation, ~55% CD31^+^CD34^+^ EPCs were obtained in the light‐induced protocol (Figure 2C,D) with a comparable efficiency (~55%^16^) to our previous small‐molecule based approach. In addition, the resulting EPCs expressed another endothelial marker VE‐cadherin (VECAD) (Figure 2D). These results demonstrated the utility of our OptoWnt system in the endothelial cell differentiation of hPSCs.

### 2.3 Light‐induced cardiac differentiation in hPSCs

**FIGURE 3:**
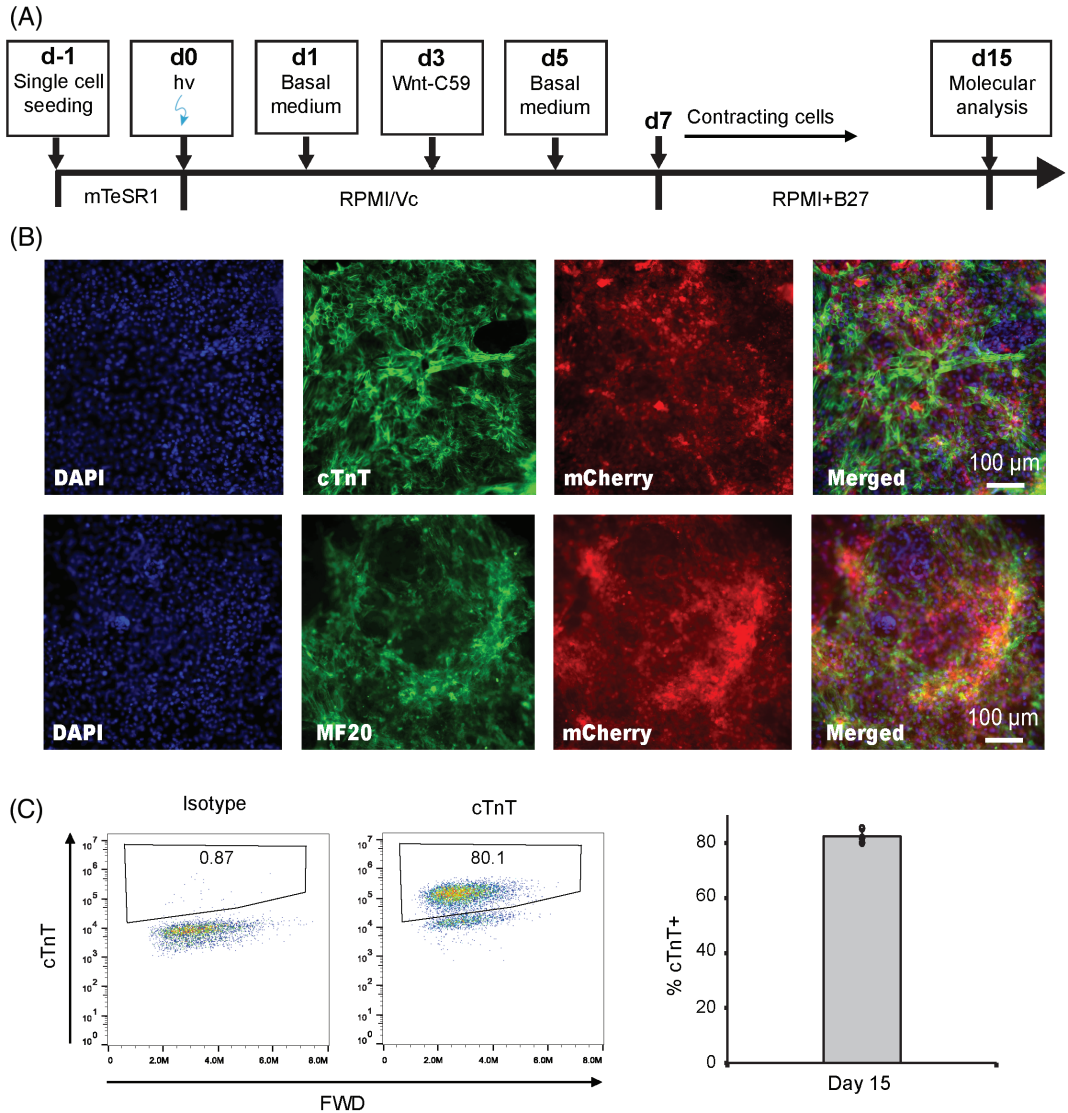
Optogenetic directed differentiation of human pluripotent stem cells (hPSCs) to cardiomyocytes. (A) Protocol for differentiation of OptoWnt hPSCs to cardiomyocytes via light‐induced activation of Wnt signaling. (B) Representative images of immunostaining for cardiac‐specific markers cTnT and MF20 as well as transgene mCherry at day 15. (C) Representative flow cytometry analysis and quantification of cTnT on day 15. Data are represented as mean ± SD of three independent replicates. Scale bars, 100 μm

To further demonstrate the utility and versatility of the OptoWnt system, we differentiated hPSCs into another mesoderm‐derived lineage − CMs − after light‐induced activation of Wnt signaling in hPSCs. We previously developed a small molecule‐based GiWi protocol to induce cardiac differentiation in hPSCs by temporal regulation of Wnt signaling, in which Gsk3β inhibitor (Gi) CHIR was used for mesoderm induction followed by treatment with Wnt inhibitor IWP2 or Wnt‐C59 to produce functional CMs. Here we used light‐induced Wnt activation to replace Gi treatment to induce mesoderm (Figure 3A), after which the illuminated cells were maintained under dark condition and treated with Wnt‐C59 to induce cardiomyocyte phenotypes.^15^ On day 15 post‐light illumination, the resulting cells expressed high levels of cardiac‐specific markers cTnT and MF20 (Figure 3B), indicating their cardiac identity. Flow cytometry analysis demonstrated that ~80% of the day 15 cells were cTnT^+^ CMs, with a differentiation efficiency (~82.7%^17^) comparable to our previous GiWi protocol.^15^

### 2.4 Optical control of spatial patterns in hPSC co‐differentiation

**FIGURE 4:**
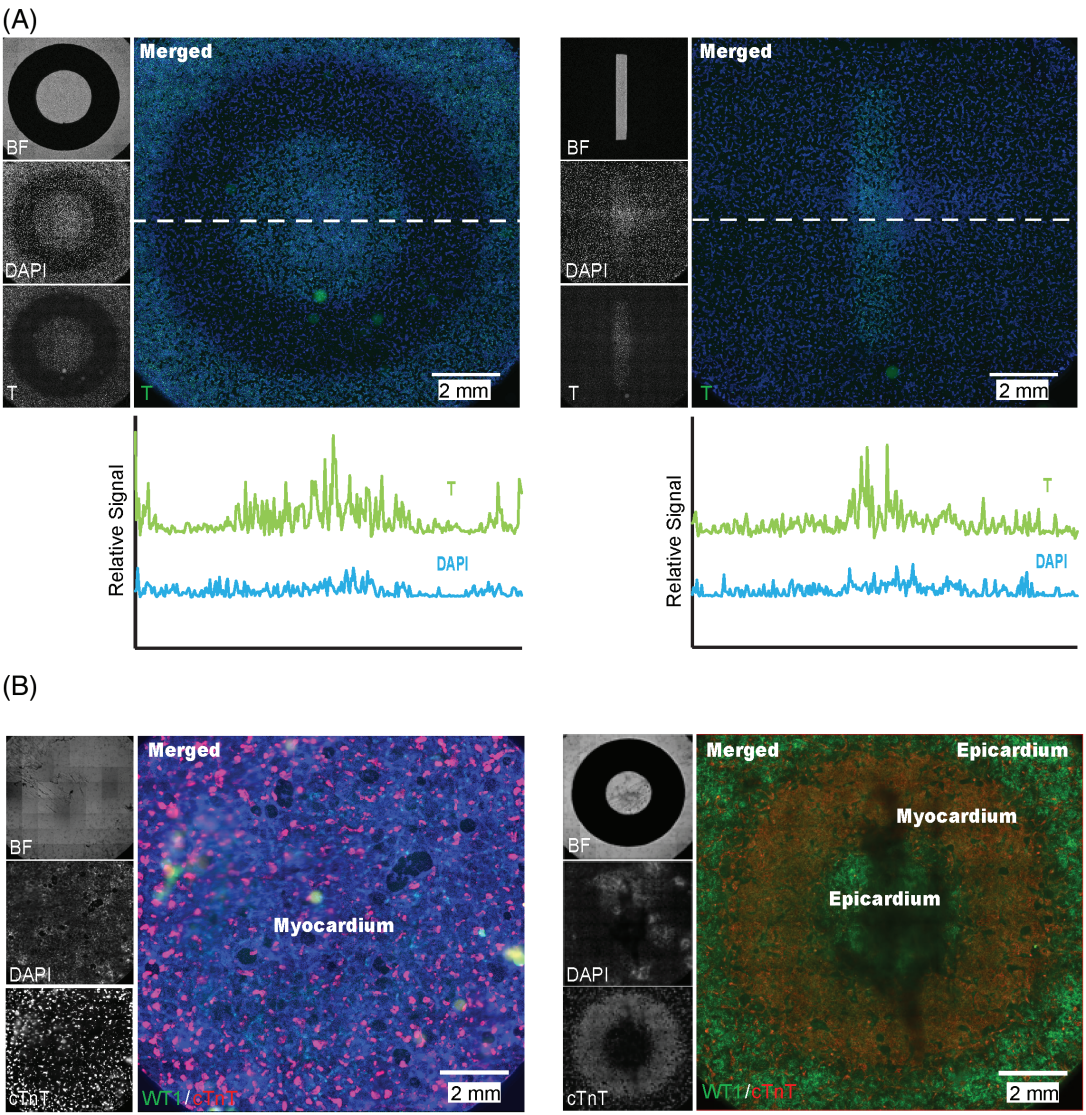
Optics‐induced spatial patterning of human pluripotent stem cell (hPSC) co‐differentiation cultures with vinyl photomasks. (A) Patterned light illumination induced T^+^ mesoderm differentiation of hPSCs via activation of Wnt signaling in unmasked regions. Left panel: ring masks; Right panel: mask with a rectangular hole. Relative expression of T and DAPI across the dashed line is shown on the bottom. (B) Patterned light illumination enabled proof‐of‐concept co‐differentiation of hPSC‐derived day 6 cardiac progenitors into cardiac and epicardial cells in a specified ring geometry of epicardium‐myocardium‐epicardium (right), as compared to a homogeneous myocardium layer with a full mask (left). Full mask was removed right before imaging. Scale bars, 2 mm

Classical cell culture methods often lack spatial control of signal dynamics to achieve custom cell patterns within heterogeneous populations, which are prerequisites of organized tissue morphogenesis and organ formation *in vivo*. To determine whether our synthetic OptoWnt platform could obtain precise spatial control of hPSC differentiation, we used custom vinyl photomasks adhered to the tissue culture plates to spatially pattern the Wnt signal.^7^, ^18^ Masks were utilized to block the light during integral periods of the cell differentiation, therefore activating or blocking the canonical Wnt pathway in desired regions with a resolution of 100 μm. The controlled admittance of Wnt signaling allows for cells within the same culture dish to be co‐differentiated into different cell types via distinct Wnt patterns. As a proof‐of‐concept, we first applied ring and rectangular masks to the OptoWnt cells and illuminated for 48 hours to induce mesoderm differentiation only at unmasked sites (Figure 4A), indicating the feasibility of using light to precisely control hPSC co‐differentiation into discrete populations. Our previous studies have demonstrated the biphasic roles of Wnt signaling in inducing hPSC differentiation into WT1^+^ epicardial and cTnT^+^ cardiac cells, two important cell types found in the distinct epicardium and myocardium layers of human heart.^10^ To reduce long‐term illumination toxicity, we applied our previous GiWi protocol to obtain day 6 cardiac progenitors from OptoWnt hPSCs, which were then seeded onto plates with vinyl photomasks for blue light illumination from day 7 to 9 and subjected for immunostaining at day 15. As compared to the homogeneous cTnT^+^ CMs under dark and homogeneous WT1^+^ epicardial cells without masks (Figure S3A), rings of light illumination resulted in structured layers of epicardium‐myocardium‐epicardium. Since Wnt signaling is critical for cardiac organoid self‐organization and chamber formation in three dimensions (3D),^19^, ^20^ we illuminated day 6 cardiac progenitor aggregates for 24 hours, leading to self‐organized cardiac organoids with a cavity (Figure S3B). Our results highlight the potential of our OptoWnt platform for light‐controllable tissue patterning in hPSCs.

## 3 DISCUSSION

In this study, we used an OptoWnt hPSC line to show that optogenetic‐mediated activation of Wnt signaling could be applied to control the spatiotemporal differentiation of hPSCs into mesoderm, endothelial and cardiac lineages via optical patterning of morphogen signaling. While the employment of illumination devices and genome editing increases the initial workload, spatial control of OptoWnt activation enables concurrent differentiation of cells into different cell types within the same dish, which is not easily achievable by traditional culture methods. Thus, we confirm the possibility of spatially controlling concurrent differentiation of hPSCs into epicardial and cardiac cells, providing a novel platform to interrogate cell‐cell interactions during tissue morphogenesis and instruct the formation of mature organ‐like structures *in vitro*. While optogenetic regulation of dominant Wnt signaling has enabled us to co‐differentiate cardiac and epicardial cells from hPSCs, spatiotemporal control of other signaling pathways, such as bFGF and VEGF, is needed to incorporate other cardiac cell types, including cardiac fibroblasts^21^ and endothelial cells,^22^ into hPSC differentiation cultures to construct spatially‐organized multicellular cardiac tissues. More recently, Wnt and BMP signaling pathways were identified as critical cues for cardiac organoid self‐organization and chamber formation in 3D, in which CMs and endothelial cells were both formed in the same aggregates.^19^, ^20^ With the advent of optogenetic control of BMP signaling,^23^ we may be able to precisely pattern the differentiation of CMs, endothelial cells and/or epicardial cells via optogenetics to achieve spatially‐organized cardiac tissues/organoids with high consistency and reproducibility. In the future, OptoWnt could be combined with 3D cell culture, orthogonal light control of other developmental signaling pathways, and advanced optical patterning techniques to achieve more physiologically relevant hPSC organoid models with intricate geometries and orthogonally controlled cell positions for studying human development and disease.

## 4 MATERIALS AND METHODS

### 4.1 Stem cell culture and genome editing

H9 wildtype hPSCs were obtained from WiCell and maintained on Matrigel‐coated 6‐well plate in mTeSR1 or mTeSR plus medium. To make OptoWnt H9 cells, Cry2‐LRP6c‐2A‐mCherry construct was cloned into AAVS1 donor plasmid (Addgene #80945) and nucleofected into H9 hPSCs along with Cas9/AAVS1 gRNA plasmid.^24^ Puromycin‐resistant mCherry+ hPSC colonies were picked and genotyped for homozygosity. The resulting OptoWnt hPSCs were maintained in dark (foil cover) and used for the downstream applications.

### 4.2 Light‐activated Wnt activation

Utilizing a LAVA device developed by Repina *et al*,^7^, ^18^ the intensity, timing, and uniformity of light exposure could be controlled for hPSC differentiation. The LAVA board was programmed to an intensity of 1 μW/mm^2^, which achieves a similar mesoderm induction to exposure to 6 μM CHIR99021. To activate Wnt, the OptoWnt cells were seeded onto Matrigel‐coated plates and placed on top of the LAVA device. If a patterned signaling was desired, the regionally transparent and regionally opaque mask was printed and placed underneath the culture dish, blocking specified regions of the cell culture dish from being exposed to the light. Both the cells and the LAVA board were placed in the tissue culture incubator at 37°C and 5% CO_2_.

### 4.3 EPC differentiation

Endothelial progenitors were obtained via a GSK‐3β inhibition or light exposure followed by an exposure to VEGF as previously reported.^16^ Briefly, mesoderm induction was achieved in OptoWnt cells either by 1‐ to 2‐day illumination of 470 nm light or by culturing with 6 μM CHIR99021 in LaSR basal medium. Starting on day 2, cells were exposed to 25 to 50 ng/mL of VEGF with medium change every 24 hours. On day 5, the cells were analyzed for flow cytometry and immunostaining of CD31, CD34, and VECAD.

### 4.4 Cardiomyocyte differentiation

In order to directly differentiate hPSCs into CMs, we followed a modified version of our previous GiWi protocol.^1^, ^2^ Mesoderm induction was achieved in OptoWnt hPSCs either by 470 nm light exposure or by culturing with 6 μM CHIR99021 in RPMI medium on day 0. On day 3, 2 μM Wnt‐C59 was used to induce cardiac differentiation in RPMI medium supplemented with 200 μg/mL ascorbic acid (AA) and 0.1% human serum albumin (HSA). On day 5, medium change with RPMI/AA/HSA. On day 7 and every 3 days afterward, medium change with RPMI/B27 medium. For co‐differentiation of cardiac and epicardial cells in Figure 4, GiWi protocol was used to obtain day 6 cardiac progenitor cells, which were then seeded onto Matrigel‐coated plates with vinyl photomasks for blue light illumination.

### 4.5 Immunostaining and flow cytometry analysis

For immunostaining analysis, cells were fixed in PBS−/− with 4% paraformaldehyde for 15 minutes and washed twice with room temperature PBS−/−. Fixed cells were then stained with appropriate primary and secondary antibodies (Table S1) in PBS−/− solution with 5% nonfat dry milk and 0.4% Triton X‐100 followed by nuclei staining. The stained cells were then imaged and processed with Leica DMi‐8 fluorescent microscope and ImageJ, respectively. Image threshold settings were held constant. For flow cytometry analysis, endothelial and cardiac cells were dissociated with TrypLE and filtered through a strainer. Singularized CMs were then fixed in 1% paraformaldehyde PBS−/− solution for 20 minutes and permeabilized in 90% cold methanol for at least an hour. After washing twice in FlowBuffer‐2 (PBS with 0.5% BSA and 0.1% Triton X‐100), CMs were stained with cTnT primary and secondary antibodies. Singularized endothelial cells were washed once with FlowBuffer‐1 (PBS with 0.5% BSA) and stained with conjugated CD31 and CD34 antibodies for 30 minutes at room temperature. Flow data was collected in a BD Accuri C6 plus machine.

## CONFLICT OF INTEREST

The authors declare no competing interests.

## AUTHOR CONTRIBUTIONS

**Conceptualization**: Yun Chang, Xiaojun Lian, Nicole A. Repina, Xiaoping Bao; **Data collection and data curation**: Peter B. Hellwarth, Yun Chang, Arundhati Das, Po‐Yu Liang, Nicole A. Repina, Xiaoping Bao; **Writing and review**: Peter B Hellwarth, Yun Chang, Arundhati Das, Po‐Yu Liang, Nicole A. Repina, Xiaojun Lian, Xiaoping Bao.

## DATA AND AVAILABILITY STATEMENT

The datasets, plasmids, and cell lines generated and used in this study are available upon request submitted to the corresponding authors.

## Supporting information


**Figure S1** Optogenetic system graphical description and apparatus.
**Figure S2**: Brachyury expression approaches saturation with increased light‐activated OptoWnt.
**Figure S3**: Light‐induced cardiac differentiation of OptoWnt hPSCs in 2D and 3D.
**Table S1**. Antibodies used in this study
