# Supplementary material for: Optogenetic‐mediated cardiovascular differentiation and patterning of human pluripotent stem cells
Source: Adv Genet (Hoboken). 2021 Sep 10;2(3):e202100011. doi: 10.1002/ggn2.202100011 (PMC9744544; doi:10.1002/ggn2.202100011)
Supplement: Supplementary file 1 — Figure S1 Optogenetic system graphical description and apparatus. Figure S2: Brachyury expression approaches saturation with increased light‐activated OptoWnt. Figure S3: Light‐induced cardiac differentiation of OptoWnt hPSCs in 2D and 3D. Table S1. Antibodies used in this study [file GGN2-2-e202100011-s001.docx]

**Supplementary Information:**

**Optogenetic-mediated cardiovascular differentiation and patterning of human pluripotent stem cells**

Peter B. Hellwarth^1,#^, Yun Chang^1,#^, Arundhati Das^1^, Po-Yu Liang^1^, Xiaojun Lian^2,*^, Nicole A. Repina^3,*^, Xiaoping Bao^1,*^

^1^Davidson School of Chemical Engineering, Purdue University Center for Cancer Research, Purdue University, West Lafayette, Indiana 47907, USA.

^2^Department of Biomedical Engineering, Huck institutes of the Life Sciences, Department of Biological, Pennsylvania State University, University Park, PA 16802, USA.

^3^Friedrich Miescher Institute for Biomedical Research (FMI), Basel, Switzerland.

^#^Co-first authors

^*^Corresponding authors: [Lian@psu.edu](mailto:Lian@psu.edu) (X.L.), [nicole.repina@fmi.ch](mailto:nicole.repina@fmi.ch) (N.A.R.), bao61@purdue.edu (X.B., lead contact)


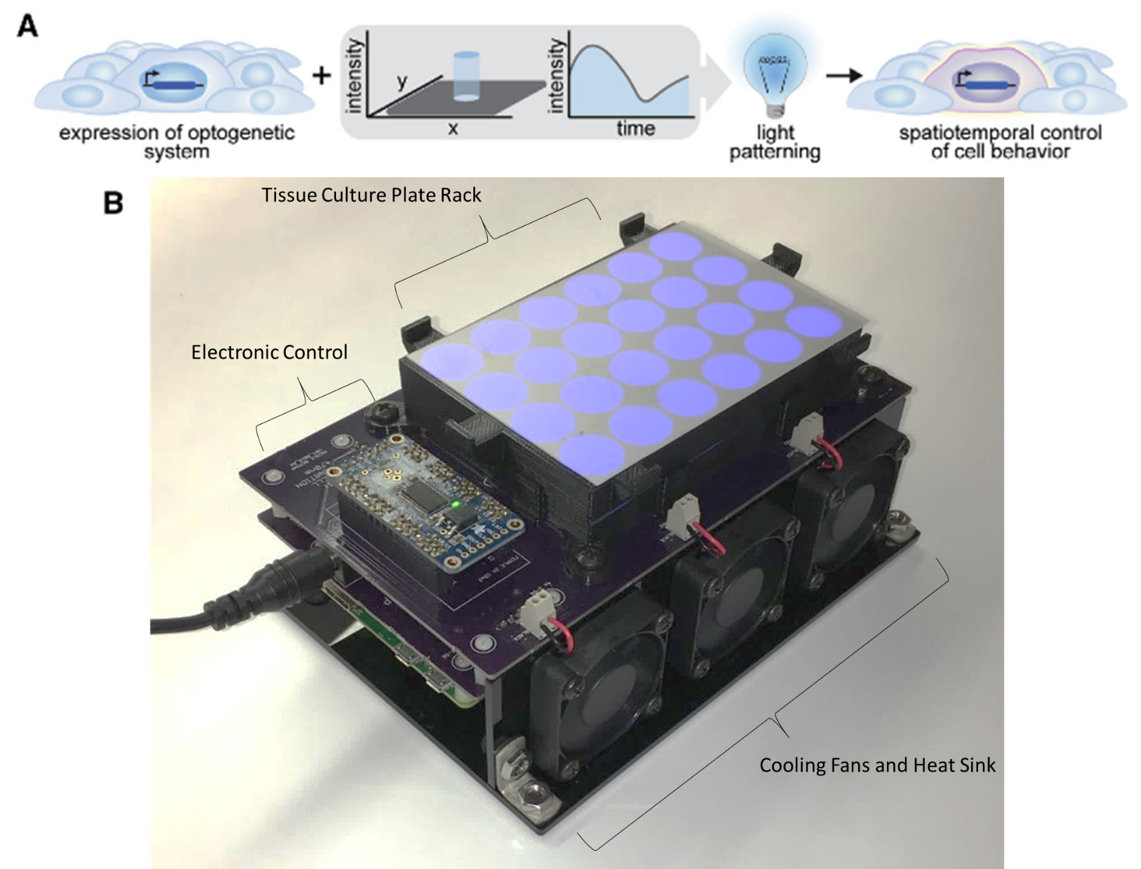


**Figure S1: Optogenetic system graphical description and apparatus**. (A) Graphical abstraction of optogenetic utilization, adapted from [8]. (B) An exploded view of the light activation at variable amplitude (LAVA) board.


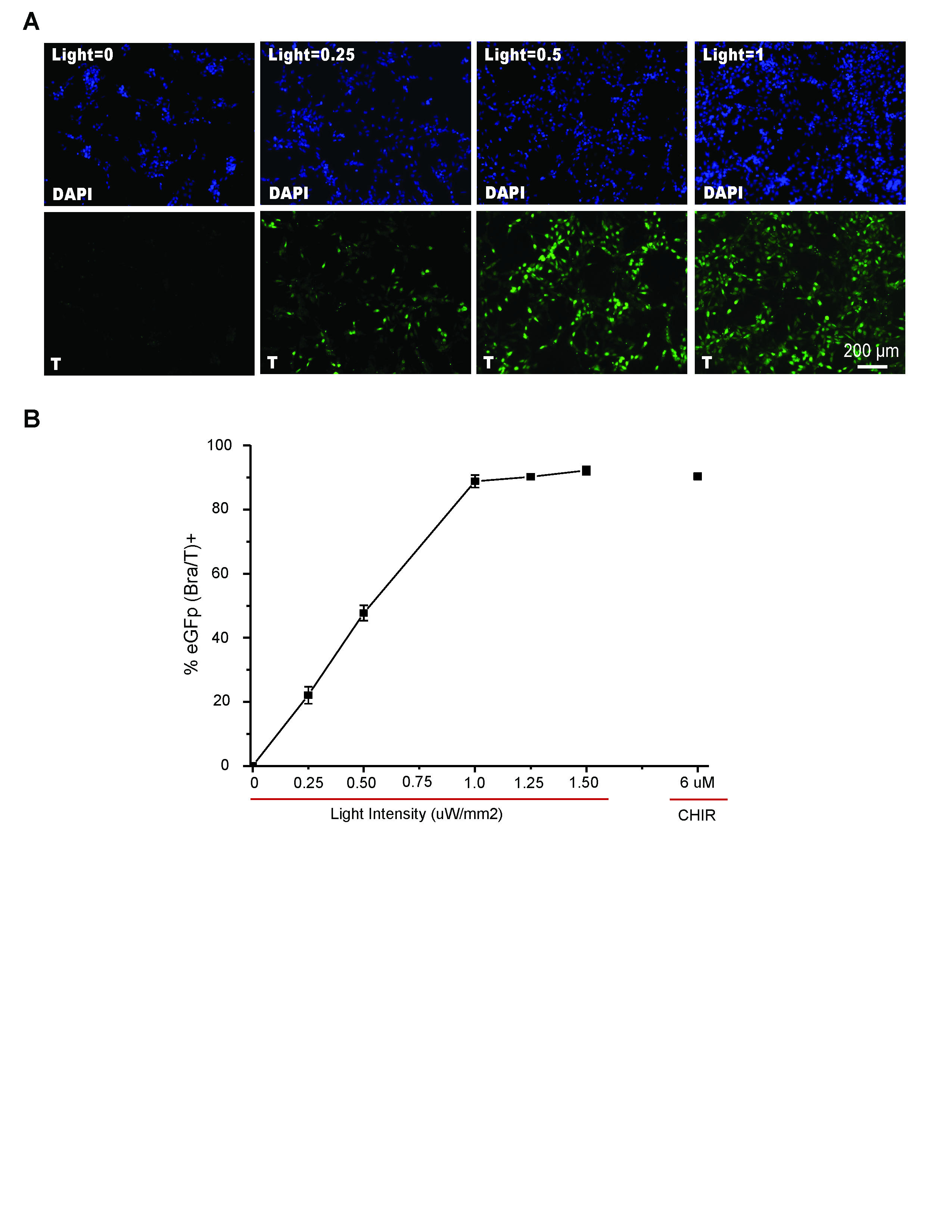


**Figure S2: Brachyury expression approaches saturation with increased light-activated OptoWnt.** With progressive increases in light intensity, cells demonstrate increases in Wnt activation, characterized by T-eGFP reporter. Representative images of immunostaining for T-eGFP (A) and quantification of GFP positive cells using flow analysis were shown in (B). Scale bars, 200 μm.


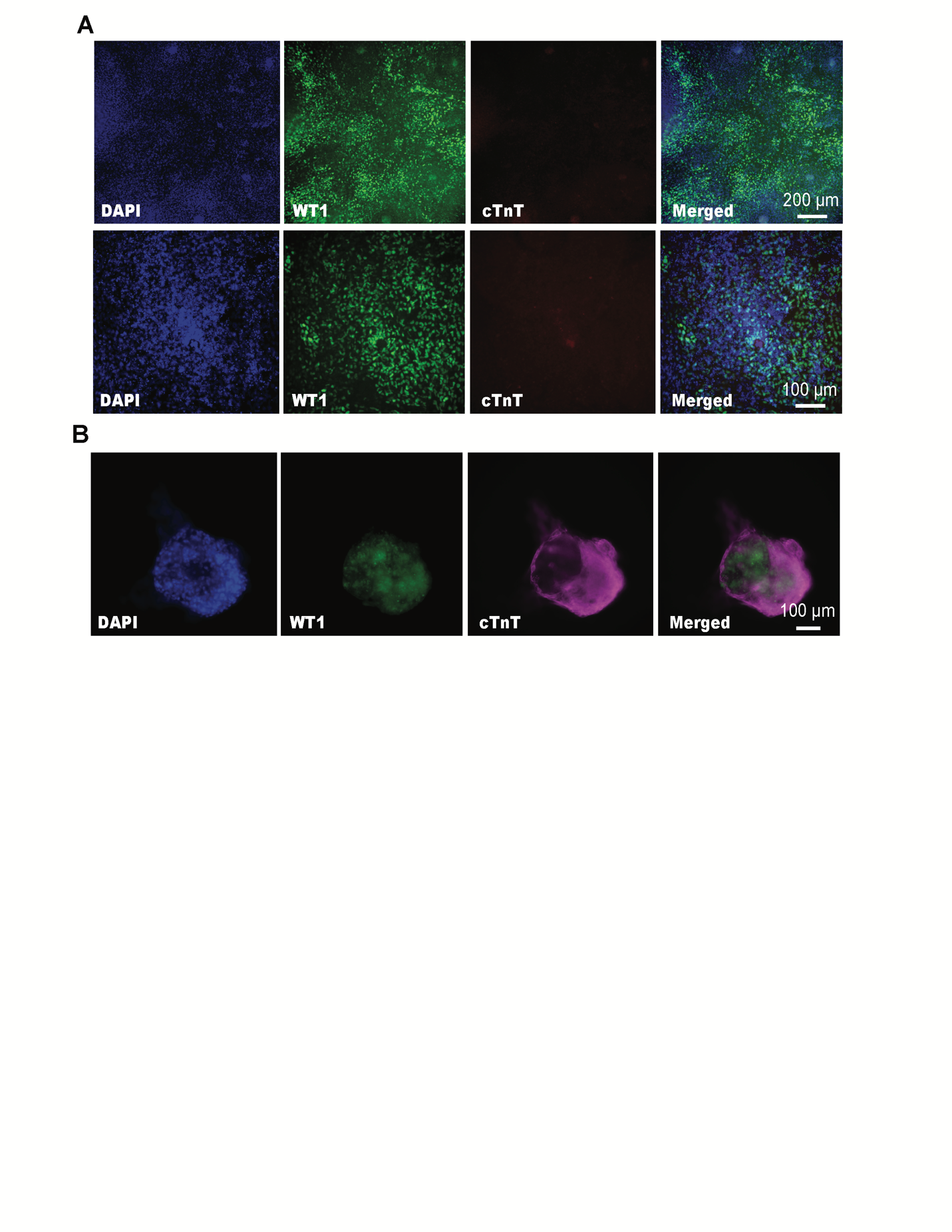


**Figure S3: Light-induced cardiac differentiation of OptoWnt hPSCs in 2D and 3D**. (A) hPSC-derived day 6 cardiac progenitors were seeded onto Matrigel-coated plates and subjected for blue light illumination without any photomask for 2 days. The illuminated cells were cultured for additional 5 days and then fixed for immunostaining. Representative images of immunostaining for WT1 and cTnT under different magnification are shown. (B) 3D day 6 cardiac aggregates were formed using hanging drop method and subjected for illumination for 24 hours. The illuminated organoids were cultured for additional 6 days before immunostaining. Representative images of immunostaining for WT1 and cTnT are shown. Scale bars, 100 or 200 μm.

Table S1

| **Antibody** | **Source/Isotype/clone /cat. no.** | **Concentration** |
| --- | --- | --- |
| OCT-3/4 | Santa Cruz/Rabbit IgG/H-134/sc-9081 | 1:100 |
| OCT-3/4 | Santa Cruz/Mouse IgG2b/C-10/sc-5279 | 1:100 |
| SSEA-4 | Santa Cruz/Mouse IgG3/813-70/sc-21704 | 1:100 |
| SOX2 | DSHB/Mouse IgG2a/PCRP-SOX2-1B3 | 1:50 |
| Brachyury | R&D Systems/ Goat polyclonal IgG/AF2085 | 1:100 |
| VE-cadherin | Santa Cruz/Mouse IgG1/F-8/sc9989 | 1:200 |
| CD31-APC | Miltenyi Biotec/Mouse IgG1/AC128/130-119-976 | 1:50 |
| CD34-FITC | Miltenyi Biotec/Mouse IgG2a/AC136/130-113-178 | 1:50 |
| cTnT | Lab Vision/Mouse IgG1/13-11/ms-295-p1 | 1:200 |
| MF20 | DSHB /Mouse IgG2b/MF20 | 1:20 |
| Secondary Antibody | Alexa 488 Goat anti-Ms IgG1/A-21121 | 1:1,000 |
| Secondary Antibody | Alexa 488 Goat anti-Rb IgG/A-11008 | 1:1,000 |
| Secondary Antibody | Alexa 594 Goat anti-Ms IgG2b/A-21145 | 1:1,000 |
| Secondary Antibody | Alexa 594 Goat anti-Ms IgG/A-21145 | 1:1,000 |
| Secondary Antibody | Alexa 594 Goat anti-Rb IgG/A-11012 | 1:1,000 |
| Secondary Antibody | Alexa 647 Goat anti-Rb IgG/A-21244 | 1:1,000 |
